# How Often Do Complications and Mortality Occur After Operatively Treated Periprosthetic Proximal and Distal Femoral Fractures? A Register-based Study

**DOI:** 10.1097/CORR.0000000000002638

**Published:** 2023-04-10

**Authors:** Simo Miettinen, Reijo Sund, Samuli Törmä, Heikki Kröger

**Affiliations:** 1 Department of Orthopaedics, Traumatology and Hand Surgery, Kuopio University Hospital, Kuopio, Finland; 2 Kuopio Musculoskeletal Research Unit, Faculty of Health Sciences, University of Eastern Finland, Kuopio, Finland; 3 Health and Social Economics Unit, Department of Health and Social Care Systems, Finnish Institute for Health and Welfare, Helsinki, Finland

## Abstract

**Background:**

The incidence of periprosthetic femoral fractures is increasing because of an increasing number of primary THAs and TKAs. High rates of complications and mortality are associated with periprosthetic fractures, but few studies have evaluated and compared the population-based incidences of these events after fractures.

**Questions/purposes:**

(1) What is the annual incidence of periprosthetic fractures treated with surgery in one hospital district in Finland? (2) How are those incidences changing over time? (3) What is the risk of complications, reoperations, and death after those injuries?

**Methods:**

This register-based study evaluated 2259 patients who underwent revision THA or TKA or any surgery for a femoral fracture between January 2004 and December 2016 at the only hospital in our district where these types of operations are performed. During the study period, the diagnosis and operation codes of the operated-on patients varied greatly, and they were somewhat inaccurate. We thus evaluated radiographs of all 2259 patients one by one, and created inclusion and exclusion criteria based on radiologic findings and medical records. Of those, 12% (279 of 2259) had periprosthetic fractures that met the inclusion criteria, and from these, we formed two study groups (periprosthetic proximal femur fractures, n = 171; periprosthetic distal femur fractures, n = 108). Eighty-eight percent (1980 of 2259) of the patients were excluded because they were treated for a condition other than periprosthetic femoral fracture. The follow-up period ended in December 2019 or at the time the patient died. To evaluate the population-based incidence, we drew the number of individuals with THA or TKA in the hospital district from the Finnish Arthroplasty Register and the Finnish Hospital Discharge Register. The characteristics of patients with operatively treated periprosthetic femoral fractures were evaluated in terms of age, gender, fracture type, implant type, and time from the index operation to periprosthetic fracture. The annual incidences of periprosthetic femoral fractures are summarized per 1000 person-years of individuals living with an implanted THA or TKA and per 100,000 individuals per year living in our hospital district. The risks of death, complications, and reoperations were evaluated for both groups, and comparisons were made in terms of patient characteristics.

**Results:**

The mean annual incidence of operatively treated periprosthetic proximal femur fractures per 1000 people living with THA implants was 2.3 ± 0.9 (95% confidence interval 1.8 to 2.7) per year, and for those with periprosthetic distal femur fractures with TKA implants, it was 1.3 ± 0.6 (95% CI 1.0 to 1.7). There was an increasing trend in the incidence of periprosthetic proximal femur fractures from 1.6 to 3.8 (95% CI 1.8 to 2.8) per 1000 arthroplasties, and it increased from 0.4 to 1.7 (95% CI 2.4 to 4.4) for periprosthetic distal femur fractures between 2004 and 2016. The mean population-based incidence of periprosthetic proximal femur fractures per 100,000 person-years was 5.3 ± 2.2 (95% CI 4.1 to 6.4) per year, and for periprosthetic distal femur fractures, it was 3.4 ± 1.7 (95% CI 2.5 to 4.4). The incidence of periprosthetic proximal femur fractures related to 100,000 person-years increased from 3.2 to 8.9 (95% CI 3.9 to 6.6), while the incidence of periprosthetic distal femur fractures increased from 1.3 to 4.4 (95% CI 2.4 to 4.8) during the study period. The cumulative incidence of major complications after periprosthetic proximal femur fracture was 8.8% at 1 year (95% CI 5.1% to 13.6%) and 12.3% at 10 years (95% CI 7.5% to 18.4%), and after periprosthetic distal femur fracture, it was 7.4% at 1 year (95% CI 3.5% to 13.4%) and 9.3% at 10 years (95% CI 4.7% to 15.7%). The cumulative incidence of reoperation after periprosthetic proximal femur fracture was 10.5% at 1 year (95% CI 6.5% to 15.7%) and 13.5% at 10 years (95% CI 8.9% to 19.1%), and for periprosthetic distal femur fracture, it was 8.3% at 1 year (95% CI 4.1% to 14.5%) and 13.8% at 10% years (95% CI 7.8% to 21.4%). The cumulative incidence of death after periprosthetic proximal femur fracture was 8.2% at 1 year (95% CI 4.7% to 12.9%) and 47.3% at 10 years (95% CI 38.1% to 55.9%), and after periprosthetic distal femur fractures, it was 14.8% at 1 year (95% CI 8.8% to 22.2%) and 67.8% at 10 years (95% CI 56.3% to 76.9%).

**Conclusion:**

The increased use of THA and TKA has led to an increase in the incidence of operatively treated periprosthetic fractures, which means there will be more revisions in the future. Older age, frailty of these patients, and often-complicated fracture patterns are related to a high rate of complications, reoperations, and mortality. Healthcare systems must prepare for a large increase in revisions for periprosthetic fracture, which are morbid events for patients and costly ones for healthcare systems.

**Level of Evidence:**

Level III, therapeutic study.

## Introduction

The increasing use of primary THAs and TKAs has been accompanied by a corresponding rise in periprosthetic femoral fractures [7, 19]. Typically, periprosthetic femoral fractures are caused by low-energy falls in patients with loose prosthetic components or osteolytic lesions [2, 22]. Increasing life expectancies among patients who undergo THA and TKA also increase the likelihood that patients with medical comorbidities will have these severe events later in life, when they are less able to tolerate them [1, 4, 5, 7]. Unsurprisingly, these injuries are associated with a high risk of complications [2, 21]. Additionally, patients who undergo primary arthroplasty are younger than those in the past, and consequently, their activity level is higher, which may predispose them to periprosthetic fractures [19].

The incidence of periprosthetic femoral fractures is increasing, and for many years, it has been the third most-common reason for THA revision, after infection and dislocation [33]. The incidence of periprosthetic fractures in patients with THA was reported to range from 0.9% to 2.1% after primary THA [3, 18, 21]. The incidence of periprosthetic distal femur fractures after TKA was reported to range from 0.4% to 2.5% for primary TKA [3, 7, 28]. However, these estimates must be considered imprecise, because the patient populations reported to date have been heterogeneous. The incidence depends on patient demographics, the number of patients with revision in the followed study group, and follow-up routines. A better approach might be to present the incidence as the annual incidence of periprosthetic femoral fractures related to patients living with arthroplasty implants per year in a specific area or country or per population of 100,000 people. In the future, this might lead to more predictable planning of service provision for patients with these fractures.

We therefore asked: (1) What is the annual incidence of periprosthetic fractures treated with surgery in one hospital district in Finland? (2) How are those incidences changing over time? (3) What is the risk of complications, reoperations, and death after those injuries?

## Patients and Methods

### Study Design and Setting

This register-based study evaluated 2259 patients who underwent revision THA or TKA or any surgery for femoral fractures between January 2004 and December 2016 at the only hospital in our district where these types of operations are performed. Patients were screened from the institutional medical records using the International Classification of Diseases, Tenth Revision (ICD-10) coding system and the Nordic Classification of Surgical Procedures operative coding system. ICD-10 codes were recorded during treatment with high variation, because codes M05.8, M16.0 to M16.7, M17.1 to M17.9, M79.6, M84.0 to M84.3, M96.6, S72.0 to S72.9, S73.0, T02.6, T80.0, T81.0, T81.4, T84.0 to T84.9, T93.1, and T93.2 were used. The operative codes NFJ60, NFJ62, NFJ64, NFJ84, NFJ86, NFJ99, NFK99, NFC20, NFC30, NFC99, NGC20, NGC30, NGC40, and NGC99 were used. We reviewed the medical records and radiographs of all patients who had any combination of these ICD-10 codes or operative codes.

The annual population of the hospital district was extracted from the national database provided by the Finnish Institute for Health and Welfare [33]. The number of patients with THA or TKA living in our hospital district at the end of each year during the study period was calculated using data for all THA and TKA reoperations since 1980 from the Finnish Arthroplasty Register and Finnish Hospital Discharge Register that were provided by the Finnish Institute for Health and Welfare [24, 32]. The annual number of primary THAs and TKAs in our hospital district was collected from the Finnish Arthroplasty Register [12]. It has been shown that accurate information can be obtained about arthroplasties in Finland by combining data from the Finnish Arthroplasty Register and the Finnish Hospital Discharge Register [34].

Because of great variance in entering operation codes into registers, further evaluation of the incidence of periprosthetic femoral fractures might be distorted. The data entered in medical registers are heterogenous in terms of operative diagnoses and operative codes [18, 19, 23]. We think this currently used study method is the most accurate way to discover the true incidence of periprosthetic femoral fractures.

The follow-up period ended on December 31, 2019, or after the death of the patient. The current living areas of the patients were confirmed at the end of the follow-up period using institutional medical records, which are connected to the Finnish Institute for Health and Welfare [32]. We assume that if the annual incidences of periprosthetic fractures in certain districts are accurately related to patients who have undergone arthroplasty, this fact can be used to estimate these incidences in general among patients undergoing arthroplasty.

### Patients’ Characteristics

**Fig. 1:**
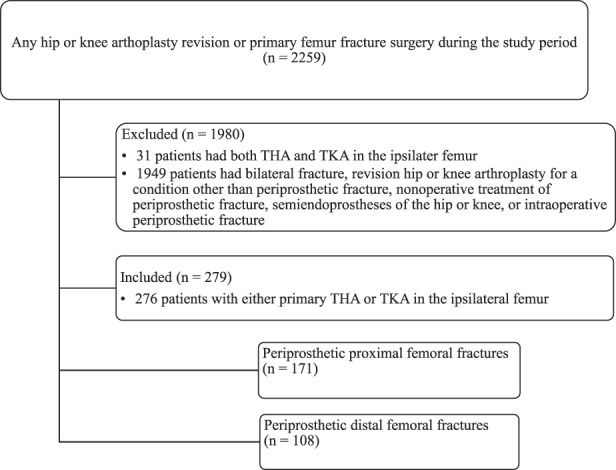
This flowchart shows the patients who were included in this study.

A total of 2259 patients were identified using the above-noted operative codes in our institution during the study period; of these, 279 periprosthetic fractures in 276 patients who underwent operative treatment for periprosthetic fractures were identified and included in this study (Fig. 1).

**Fig. 2:**
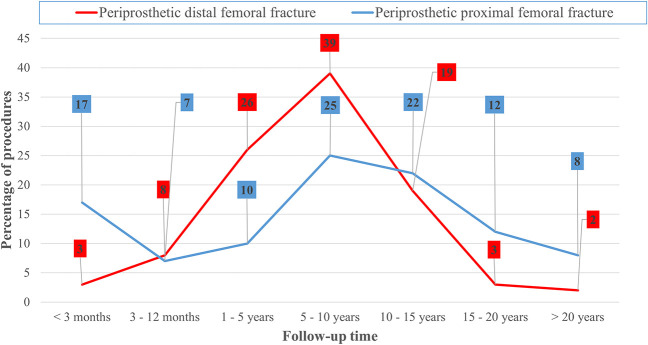
The time from the index arthroplasty to surgery for periprosthetic proximal femoral fractures (n = 153) and periprosthetic distal femoral fractures (n = 62) is shown. The timing of the index THA was missing for 18 patients and the timing of TKA was missing for 46 patients. All of these patients underwent surgery before 2004 and were excluded. A color image accompanies the online version of this article.

The mean follow-up time was 5.0 ± 3.6 years (range 4 days to 16 years). The mean time from the primary THA to proximal periprosthetic fracture was 8.8 ± 6.9 years (range 4 days to 26 years), and from the primary TKA to distal periprosthetic fracture, it was 7.1 ± 4.8 years (range 0.2 to 26 years; p = 0.53) (Fig. 2).

### Variables

Patient characteristics (gender, age, operative side, implant type, fracture type, time of primary arthroplasty, and postoperative complications) were collected from the institutional medical records for between-group comparison (Table 1). We performed radiologic analyses using AP and lateral pelvic radiographs.

**Table 1.** Characteristics of operatively treated patients with periprosthetic femoral fractures

Parameter	Periprosthetic proximal femoral fracture (n = 171)	Periprosthetic distal femoral fracture (n = 108)	p value
Female sex, % (n)	58 (99)	86 (93)	< 0.001
Operative side, right, % (n)	55 (94)	54 (58)	0.84
Age in years, mean ± SD	75 ± 11	80 ± 9	< 0.001
Female patients, mean ± SD	75 ± 11	81 ± 8	< 0.001
Male patients, mean ± SD	75 ± 12	72 ± 13	0.49
Age ≥ 75 years, % (n)	57 (98)	75 (81)	0.003
Femur implant type, % (n)			
Cementless	78 (133)		
Cemented	22 (38)	100 (108)	
Fracture classification, % (n)			
Rorabeck classification			
Type II		95 (103)	
Type III		5 (5)	
Vancouver classification			
Type A			
AG, greater trochanter	8 (13)		
AL, lesser trochanter	8 (13)		
Type B			
B1, stem well fixed	37 (64)		
B2, stem loose	20 (35)		
B3, stem loose, poor bone stock	14 (24)		
Type C, well below stem	13 (22)		
Fracture fixation method, % (n)			
Lateral plating	63 (107)	94 (102)	
Femoral component revision	30 (51)		
Cerclage wiring	8 (13)		
Tumor prosthesis		3 (3)	
Femur amputation		2 (2)	
Femoral retrograde nailing		1 (1)	

Data presented as % (n) or mean ± SD.

Major complications after periprosthetic fractures were defined as prosthesis dislocation, nonunion, deep infection, component loosening, another fracture, nerve damage, serious implant irritation, and other serious events. We analyzed major complications with and without revision surgery separately. Other complications, including superficial infection, postoperative hematoma, delayed union, and deep venous thrombosis that did not result in a serious event or reoperation were considered minor.

We classified periprosthetic fractures according to the Vancouver classification system [8]. The Vancouver classification system considers the location of the fracture relative to the stem, the stability of the implant, and the associated bone loss [8]. We classified periprosthetic distal femoral fractures according to the Rorabeck classification system, where Type I is a nondisplaced fracture where the femoral component is intact. In Rorabeck Type II, the fracture is displaced, and the femoral component is fixed. Rorabeck Type III refers to a displaced fracture and loose femoral component [27]. Two authors (SM and ST) classified the fractures using medical records and radiographs. The surgeon determined the stability of the periprosthetic fracture intraoperatively. Additionally, the surgeon selected the fracture fixation method before or during the operation based on the fracture pattern and implant stability. The primary operation method used was lateral plating, sometimes with cerclage wiring or strut grafts. Other methods were multiple cerclage wires or revision of the loosened femoral component. In some patients, plating, cerclage wiring, or strut grafts were included in the implant revision, if needed. In five patients with distal periprosthetic fractures, tumor prosthesis implantation (two patients), femoral amputation because of the poor medical condition of the patient (two patients), and retrograde nailing (one patient) were performed.

### Primary and Secondary Study Outcomes

Our primary study goal was to evaluate the annual incidence of periprosthetic fracture per 1000 person-years among people living in the institutional district during the study period who had a primary THA or TKA implant, using the institutional medical records.

Our secondary study goals were to evaluate the annual incidence of primary arthroplasties and periprosthetic fractures per 100,000 inhabitants per year living in our hospital district, how those incidences are changing over time, the time from the index arthroplasty to periprosthetic fracture, and the risk of death, complications, and reoperations after those injuries.

### Ethical Approval

Ethical approval for this study was obtained from the institutional review board of Kuopio University Hospital (103/2019).

### Statistical Analysis

Continuous data were compared using the Mann-Whitney U test. For categorical data, we used the chi-square test. We used a cumulative incidence function analysis, considering competing risks, to study death, postoperative complications, and reoperations. All p values < 0.05 were considered statistically significant. The data were analyzed using SPSS (IBM Corp) and R (R Foundation for Statistical Computing, version 3.6.2).

## Results

### Annual Incidence of Periprosthetic Femoral Fracture and Fractures Undergoing Surgical Treatment

The mean annual incidence of periprosthetic proximal femoral fractures was 2.3 ± 0.9 (95% CI 1.8 to 2.7) per 1000 individuals with THA, and it was 1.3 ± 0.6 (95% CI 1.0 to 1.7) per 1000 individuals with TKA treated for distal femoral fractures. The mean population-based incidence of periprosthetic proximal femur fractures related to 100,000 person-years was 5.3 ± 2.2 (95% CI 4.1 to 6.4) per year and 3.4 ± 1.7 (95% CI 2.5 to 4.4) per year for distal periprosthetic femur fractures.

### Changes in Incidence Over Time

The change in the annual incidence of periprosthetic proximal femoral fractures per 1000 individuals with arthroplasty was 1.6 to 3.8 (95% CI 1.8 to 2.8) and 0.4 to 1.7 (95% CI 2.4 to 4.4) for distal femoral fractures. The incidence of periprosthetic proximal femur fractures per 100,000 person-years increased from 3.2 to 8.9 (95% CI 3.9 to 6.6), while the incidence of periprosthetic distal femur fractures increased from 1.3 to 4.4 (95% CI 2.4 to 4.8) during the study period.

**Fig. 3:**
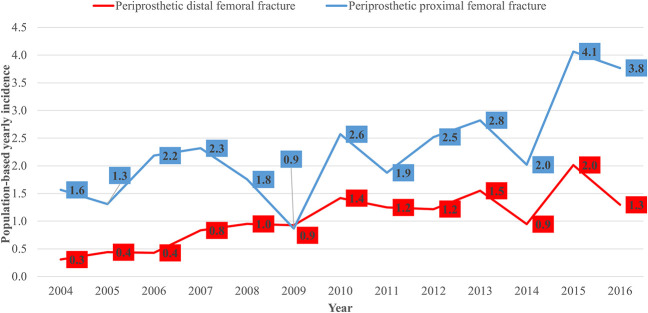
This figure shows the population-based yearly incidence of periprosthetic femoral fractures related to 1000 individuals living with THA or TKA implants in our hospital district. A color image accompanies the online version of this article.

In 2004, there were 1.6 periprosthetic proximal femur fractures and 0.3 periprosthetic distal femur fractures per 1000 individuals living with arthroplasty, whereas in 2016, the annual incidences increased to 3.8 periprosthetic proximal femur fractures (+ 240%) and 1.3 periprosthetic distal femur fractures (+ 430%) per 1000 individuals (Fig. 3). During the same time, the number of patients living with THA was 5110 in 2004, and in 2016, it was 5840 (+ 14%). Additionally, for TKA, it was 6491 in 2004, and in 2016, it was 8485 (131%). Meanwhile, the mean population-based incidence of primary THA was 192 ± 25 per 100,000 person-years (95% CI 179.7 to 205.7), and for TKA, it was 217 ± 47 per 100,000 person-years (95% CI 195.2 to 243.4) during the study period at our institution. The number of patients undergoing primary THA increased by 13.7% (from 2035 to 2357) during the study period, and the number of patients having TKA increased by 16.9% (from 5344 to 6432).

### Risk of Complications, Reoperations, and Death After Fracture

The mean time to complications was 1.0 ± 1.9 years (95% CI 0.8 to 2.8 years) for periprosthetic proximal femur fractures and 1.1 ± 1.7 years (95% CI 0.6 to 2.4 years) for periprosthetic distal femur fractures (p = 0.14). The cumulative incidence of one or more major complications (defined as dislocation, nonunion, deep infection, component loosening, and other serious events such as new fracture because of trauma, nerve damage, or serious implant irritation) (Table 2) within 1 year of the injury was 8.8% (95% CI 5.1% to 13.6%), 10.0% (95% CI 6.0% to 15.1%) within 5 years, and 12.3% (95% CI 7.5% to 18.4%) within 10 years after surgery for periprosthetic proximal femoral fractures, and it was 7.4% (95% CI 3.5% to 13.4%) within 1 year and 9.3% (95% CI 4.7% to 15.7%) within 5 and 10 years, using a competing risks estimator.

**Table 2.** Cumulative incidence of major complications after operatively treated periprosthetic femoral fractures

	Periprosthetic proximal femoral fracture (n = 171), %						Periprosthetic distal femoral fracture (n = 108), %					
Major complication type	1 year	95% CI	5 years	95% CI	10 years	95% CI	1 year	95% CI	5 years	95% CI	10 years	95% CI
Prosthesis dislocation	5.3	2.6 to 9.3	6.5	3.4 to 10.9	6.5	3.4 to 10.9						
Deep infection	4.8	2.2 to 8.6	5.3	2.6 to 9.3	6.7	3.2 to 11.9	5.6	2.3 to 11.0	7.4	3.5 to 13.4	7.4	3.5 to 13.4
Nonunion of the fracture	1.1	0.2 to 3.8	2.9	1.1 to 6.3	2.9	1.1 to 6.3	5.6	2.3 to 11.0	8.3	4.1 to 14.5	8.3	4.1 to 14.5
Loosening of any component	3.5	1.4 to 7.1	4.1	1.8 to 7.8	4.1	1.8 to 7.8						
Other	4.7	2.2 to 8.6	4.7	2.2 to 8.6	5.6	2.7 to 9.9	3.7	1.2 to 8.6	4.9	1.7 to 10.4	6.3	2.5 to 12.7

The cumulative incidence of reoperation within 1 year of injury was 10.5% (95% CI 6.5% to 15.7%) and 13.5% (95% CI 8.9% to 19.1%) within 5 and 10 years after surgery for periprosthetic proximal femoral fractures, and it was 8.3% (95% CI 4.1% to 14.5%) within 1 year, 12.2% (95% CI 6.9% to 19.4%) within 5 years, and 13.8% (95% CI 7.8% to 21.4%) within 10 years for periprosthetic distal femur fracture.

**Fig. 4:**
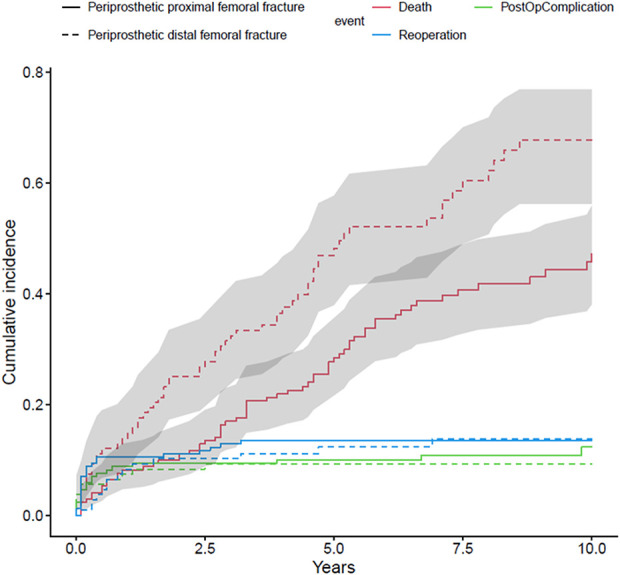
This figure shows the cumulative incidence for complications (green line), reoperations (blue line), and death (red line) after surgery for a periprosthetic femoral fracture in case of proximal (solid line) and distal fractures (dashed line). Principle 2. A color image accompanies the online version of this article.

The cumulative incidence of death within 30 days was 2.9% (95% CI 1.1% to 6.3%), 5.3% (95% CI 2.6% to 9.3%) within 90 days, 8.2% (95% CI 4.7% to 12.9%) within 1 year, 11.1% (95% CI 6.9% to 16.3%) within 2 years, 28.4% (95% CI 21.6% to 35.6%) within 5 years, and 47.3% (95% CI 38.1% to 55.9%) within 10 years of injury for periprosthetic proximal femoral fractures. For periprosthetic distal femoral fractures, it was 7.4% (95% CI 3.5% to 13.3%) within 30 days, 12.0% (95% CI 6.8% to 19.0%) within 90 days, 14.8% (95% CI 8.8% to 22.2%) within 1 year, 25.0% (95% CI 17.3% to 33.5%) within 2 years, 48.2% (95% CI 37.9% to 57.7%) within 5 years, and 67.8% (95% CI 56.3% to 76.9%) within 10 years (Fig. 4). The mean age of patients with periprosthetic distal femur fractures was 83 ± 7.8 years (range 55 to 95 years). Among patients with periprosthetic proximal femur fractures, the mean age was 79 ± 9.5 years (range 49 to 95 years) (p = 0.01).

## Discussion

The increasing number of periprosthetic fractures has been recognized for more than two decades [7, 18]. However, the true incidence of these fractures among individuals with arthroplasty and in the general population is unknown. When these population-based incidences are known better, we might predict the future numbers of periprosthetic fractures to provide services. Typically, these fractures affect patients who are older and who have medical comorbidities that result in decreased physiologic reserve to recover from trauma or revision surgery [26, 29]. If risk factors for proximal and distal periprosthetic fractures are better known, we will be better positioned to prepare our healthcare systems to treat them effectively. We found an upward trend in the population-based incidence of operatively treated proximal and distal periprosthetic fractures during the 12-year study period, even as the number of individuals with THA or TKA rose less steeply, which shows that the incidence of these fractures and the revisions they will cause represent a real demographic concern.

### Limitations

There might be selection bias, because patients were retrospectively evaluated from a large patient series, and only operatively treated patients were included. However, we think this selection method is not a problem because most of the periprosthetic fractures were operatively treated. Further, there is a risk that some patients might have had some other operative codes than those used in this study, and therefore they would not have been included. To limit this possible selection bias, we included as many diagnostic and operational codes as we think were relevant for retrospective medical database searching. In addition, if patients were treated elsewhere, they may have not been recorded in our institution’s medical records. However, we think our study protocol would have identified these patients because they would have appeared in our institution’s medical record when these patients returned for follow-up radiographs later, because follow-up occurs by district in Finland. Comparisons of survival and complications between the groups have some limitations, because there were few comparative demographic data regarding patients with these injuries available to us. Nonetheless, we think the data of the study groups are comprehensive enough to give a nuanced view of the key differences between the groups in terms of risk factors. Another weakness is that the treatment of periprosthetic fractures has changed over time; however, presumably, the same would occur in other settings during the study period. Thus, our findings ought to generalize reasonably well. Additionally, patient-reported outcomes were not available, and our main endpoints were complications, reoperations, and death. We believe we followed these patients long enough to identify the degree to which those endpoints could fairly be attributed to the fracture that triggered study inclusion.

### Annual Incidence of Periprosthetic Femoral Fracture and Fractures Undergoing Surgical Treatment

The annual incidence of periprosthetic femoral fractures increased in one hospital district in Finland, and a high proportion of these patients underwent major surgery to treat these injuries. The current study results agree with a recent study from Sweden in which the incidence of periprosthetic femoral fractures increased from 1.0 to 1.4 per 1000 primary THAs between 2001 and 2011 [5]. The cumulative probability of proximal periprosthetic fracture was 0.4% at 1 year, 0.8% at 5 years, 1.6% at 10 years, and 3.5% at 20 years [2]. Distal periprosthetic fractures have been reported to occur in 0.2% to 1.8% of patients after primary TKA [3, 21, 28]. There is a relative lack of studies examining the annual incidence of operatively treated proximal periprosthetic fractures in people with THA implants and in the general population living in well-defined hospital districts or geographic regions. Usually, this incidence is typically given as a percentage related to the number of primary THAs in a single center or is based on large national register data with a variable timescale [18, 23]. The incidence of periprosthetic proximal fractures undergoing surgery was 5.3 per 100,000 person-years, and it was 3.2 per 100,000 person-years for periprosthetic distal fractures. We could not find any similar studies with periprosthetic fractures, but interestingly, our study findings agree with the previously reported annual incidences of distal femoral fractures without arthroplasty, which have varied from 2.4 to 8.7 per 100,000 person-years [6, 9].

### Changes in Incidence Over Time

These serious injuries appear to be becoming much more common over time. Frenzel et al. [13] had similar results in their institution; they found that the incidence of periprosthetic femur fractures has increased 2.5-fold over the past two decades, and the risk of periprosthetic fracture was higher after THA than after TKA. Our findings support those results, because we found that the annual incidence of periprosthetic fractures increased three to four times more quickly than the total number of patients with endoprostheses.

There seem to be two postoperative peaks for periprosthetic femoral fractures, the first occurring shortly after surgery, and the second peak occurring 5 to 15 years later. In our study, 10% of all periprosthetic femoral fractures occurred during the early postoperative stage, which we considered to be the 3 months after the index THA. Late postoperative periprosthetic femoral fractures are frequently associated with loosening or osteolysis of the femur component [20, 30]. In contrast, distal periprosthetic femoral fractures seem to have one peak, occurring between 5 and 10 years after the primary TKA. In this study, the mean time to distal periprosthetic femoral fracture was 7 years after the index TKA, which is similar to the finding of 9.5 years by Ross et al. [29]. Our study showed that the incidence of periprosthetic femoral fractures decreases approximately 15 years after the index arthroplasty.

### Risk of Complications, Reoperations, and Death After Fracture

Complications, reoperations, and death after periprosthetic femoral fracture occurs with disconcerting frequency. We found a major complication rate of 17%, which supports previous complication rates of even greater than 20% [10, 14]. Most of the major complications were in patients with proximal periprosthetic femoral fractures. The most common complication was prosthesis dislocation with a rate of 6%, which is lower than in previous studies (11% to 16%) [1, 23]. The second-most common complication in the periprosthetic proximal femoral fracture group and the most common complication in the periprosthetic distal femoral fracture group was deep infection, with a proportion of 5% in both groups. This study finding is similar to previous evidence reporting postoperative periprosthetic femoral fracture infection rates varying from 3% to 13% [15, 16, 26]. Nonunion was the second-most common major complication in the periprosthetic distal femoral fracture group with a rate of 5%, which is somewhat lower than in other similar studies, where it varied from 7% to 16% [15, 16, 26, 29]. However, the true complication rate of periprosthetic femoral fractures is difficult to compare with that of other studies of the same type because the patient populations reported elsewhere are heterogeneous, and there are many factors (such as patient demographics, follow-up time, the use of implants, surgical technique, and follow-up routines) that may affect the results [14, 16, 17].

Overall, 60% of the patients with periprosthetic femoral fractures died during follow-up, and there was a difference between the groups, because the mortality rate of the periprosthetic distal femoral fracture group was 68%, whereas the rate of the periprosthetic proximal femoral fracture group was lower, at 54%. Similarly, 1-year mortality rates after periprosthetic proximal femoral fracture vary from 10% to 18% [11, 19, 30]. After periprosthetic distal femoral fracture, 1-year mortality rates of 11% to 15% have been reported [10, 17, 29], and these findings agree with our study. Notably, in this study, patients with distal periprosthetic femoral fractures were older than patients in the proximal periprosthetic femoral fracture group, and this may have resulted in the greater mortality in that group. These findings generally support those of Shields et al. [31], who found that the mortality of patients with periprosthetic fracture increases among older patients. This study showed that among the patients with periprosthetic fracture who died, there were more women in the periprosthetic distal fracture group than in the proximal periprosthetic fracture group. These findings support previous studies; periprosthetic proximal femoral fractures, especially Vancouver Type C fractures, display typical fragility fracture characteristics, and this type is associated with female sex and high mortality [5, 17]. Moreover, Powell-Bowns et al. [25] showed that the mortality of patients with Vancouver Type C fractures was higher than those with Vancouver Type B fractures, which also agrees with our study findings. Interestingly, previous studies have shown that male patients with periprosthetic proximal fracture have a higher risk of death than women do [10, 17]. In this current study, patients with periprosthetic fracture were mostly older women in both groups, and because of this, there was no difference between the sexes in terms of survival. Direct comparisons of mortality rates among differing studies are difficult, because studies included different fracture patterns, different treatment modalities, a variety of age groups, and a variety of follow-up durations.

## Conclusion

This country-specific retrospective study showed a steeply increasing trend in the incidence of operatively treated periprosthetic fractures. High rates of complications and reoperations were found, and the reason for this seems to be that patients experiencing periprosthetic fractures are older and very likely frailer. A high rate of death is expected after periprosthetic distal fracture because these patients are disproportionately older. Healthcare systems must prepare for a large increase in revisions for periprosthetic fracture, which are morbid events to patients and costly ones for healthcare systems. More studies are needed to determine what surgical factors and postoperative rehabilitation methods might decrease the risk of complications and death after these serious injuries.
